# The Effect of Virtual Reality–Based Social Cognitive Training for Autistic Adults: Protocol for STEPS (Social Cognitive Training Enhancing Pro-Functional Skills) Randomized Clinical Trial

**DOI:** 10.2196/72854

**Published:** 2026-01-05

**Authors:** Johannes Andresen, Alberte C E Jeppesen, Anne Sofie Due, Lise Sandvig Mariegaard, Rizwan Parvaiz, Carsten Hjorthøj, Amy Elizabeth Pinkham, Merete Nordentoft, Gasper Letnar, Jens Richardt Møllegaard Jepsen, Louise Birkedal Glenthøj

**Affiliations:** 1 VIRTU Research Group, Mental Health Center Copenhagen, Copenhagen University Hospital – Mental Health Services CPH, Gentofte Hospitalsvej 3A, 4th Floor, Copenhagen, 2900, Denmark, +45 21452889; 2 Department of Psychology, University of Copenhagen, Copenhagen, Denmark; 3 Department of ADHD and Autism, Mental Health Center Glostrup, Copenhagen University Hospital – Mental Health Services CPH, Copenhagen, Denmark; 4 Copenhagen Research Center for Mental Health (CORE), Mental Health Center Copenhagen, Copenhagen University Hospital – Mental Health Services CPH, Copenhagen, Denmark; 5 Department of Public Health, University of Copenhagen, Copenhagen, Denmark; 6 School of Behavioural and Brain Sciences, The University of Texas at Dallas, Richardson, TX, United States; 7 Department of Clinical Medicine, University of Copenhagen, Copenhagen, Denmark; 8 Sanos ApS, Søborg, Denmark; 9 Child and Adolescent Mental Health Center, Copenhagen University Hospital – Mental Health Services CPH, Copenhagen, Denmark; 10 Center for Neuropsychiatric Schizophrenia Research, Mental Health Center Glostrup, Copenhagen University Hospital – Mental Health Services CPH, Copenhagen, Denmark

**Keywords:** autism spectrum disorder, virtual reality, psychosocial functioning, social skills, social cognitive training, emotion recognition, theory of mind

## Abstract

**Background:**

Autistic adults constitute a growing and largely overlooked population with limited clinical and research resources. Social cognitive impairments are key deficits faced by this population, significantly impacting social interactions, educational and vocational functioning, and quality of life. Interventions targeting social cognition in autistic adults have shown promising results. Recent studies investigating the effect of virtual reality (VR)–based interventions for autistic adults have provided preliminary evidence supporting the feasibility and effectiveness of using this innovative technology. These studies indicate that VR interventions can enhance functional and social skills and improve specific neurocognitive and social cognitive functions. However, large-scale randomized clinical trials are urgently needed to fully assess the effectiveness of VR-based interventions for autistic adults.

**Objective:**

This protocol aims to provide a comprehensive description of the design and methodology of the STEPS (Social Cognitive Training Enhancing Pro-Functional Skills) trial.

**Methods:**

STEPS is a clinical, randomized, assessor-blinded, parallel-group superiority trial. A total of 140 participants will be allocated to receive either virtual reality–based social cognitive training (VRSCT) + treatment as usual (TAU) or TAU alone. The experimental group will receive 12 weekly 1-hour sessions of VRSCT, aiming at improving psychosocial functioning and social cognition through exposure to virtual social environments. The intervention comprises 3 core modules, namely emotions, social understanding, and complex social interactions. The exact content and duration of TAU received by each participant will be mapped and documented upon trial completion. Assessments will be conducted at baseline, at cessation of the intervention (3 months post baseline), and at 6 months post baseline.

**Results:**

Participant enrollment began in May 2024. As of February 2025 (initial manuscript submission), 34 participants had been enrolled, increasing to 97 participants as of December 2025. Completion of enrollment is expected in April 2026. Data analysis is expected to begin in October 2026 following the final 6-month follow-up assessment. Results are anticipated in December 2026 and will be disseminated through peer-reviewed publications.

**Conclusions:**

To our knowledge, STEPS is the hitherto largest randomized clinical trial globally investigating the effect of VRSCT for autistic adults. The results of this innovative intervention approach may significantly advance research in the field of autism. VRSCT holds potential to improve psychosocial functioning, quality of life, and co-occurring clinical symptoms, and to reduce social cognitive deficits in autistic adults. Establishing evidence-based interventions is crucial for addressing the debilitating psychosocial challenges faced by this population, especially considering the absence of established gold-standard treatments.

## Introduction

### Terminology

In this paper, we use the term autistic adults, in line with international recommendations [1] to refer to adults on the autism spectrum. Additionally, we use the term autism spectrum condition as an equivalent to autism spectrum disorder to minimize discriminatory language.

### Autism

According to the diagnostic criteria described in the *DSM-5-TR* (*Diagnostic and Statistical Manual of Mental Disorders, Fifth Edition, Text Revision*) by the American Psychiatric Association, autism is defined as a neurodevelopmental condition characterized by persistent deficits in social communication, such as difficulties with socioemotional reciprocity, communication, and the development, maintenance, and understanding of relationships. This is accompanied by restricted, repetitive patterns of behavior, interest, or activities. These clinical characteristics must be present from early development (but may not become fully manifest until social demands exceed limited capacities or may be masked by learned strategies in later life), cause significant impairment in psychosocial functioning, and cannot be better explained by other intellectual or developmental conditions [2]. Autism is a lifelong neurodevelopmental variation that is estimated to occur in 1%‐2% of the global population [3]. The prevalence rate is increasing [4,5], as well as the lifelong economic impact associated with the condition. In 2019, the estimated lifetime social cost of an individual on the autism spectrum was approximately US $3.6 million in the United States, with the largest costs associated with lost productivity and adult care [6]. These costs should not be attributed to individuals on the autism spectrum but should rather be regarded as a consequence of inadequate support and accommodations in educational and occupational settings and a lack of effective interventions that address the specific challenges faced by the autistic population. These obstacles are particularly pronounced for autistic adults, as many experience a lack of appropriate interventions and support [7], are vastly underrepresented in the workforce, and are at heightened risk of depression and suicidal actions compared to the general population [8-11]. Despite the critical need for interventions and support systems aimed at improving outcomes for autistic adults, research on autism has primarily focused on children and adolescents, overlooking the growing population of adults who face extensive unmet treatment needs [12].

### Social Cognition in Autistic Adults

Social cognition can be defined as “(…) the mental operations that underlie social interactions, including perceiving, interpreting, and generating responses to the intentions, dispositions, and behaviors of others.” [13]. As this definition implies, social cognition is a multifaceted and complex construct, underpinned by different functions, such as emotion processing and recognition, theory of mind (ToM), empathy, social perception, social attribution, and social awareness. It is well established that autistic adults, on average, show reduced performance on social cognitive tasks, including facial emotion processing, prosody interpretation, mental state labeling, social perception, social attribution, and social understanding, compared to neurotypicals. However, social cognitive profiles in autism present differently from person to person, and individual profiles can be dynamic and may shift over time [14].

Social cognitive impairments are key deficits faced by autistic adults [15], which severely impact social interactions, hinder educational and vocational functioning, and compromise quality of life [16-18]. The social cognitive deficits in autism encompass both the more basic functions, such as recognizing emotions or making appropriate eye contact, and higher-order functions, such as inferring others’ mental states (ToM) or understanding subtle aspects of social communication [19,20]. These functions are vital to understanding and reacting adequately to dynamic, complex social situations in all aspects of life, from making friends and building intimate relationships in private life to being able to participate appropriately in educational or professional settings.

### Interventions Targeting Social Cognitive Impairments

Targeting social cognitive deficits in autistic adults without using virtual reality (VR) technology has been shown to improve psychosocial functioning [21]. These interventions often use computer- or group-based exercises to improve different aspects of social cognition (eg, perception of social cues, emotion recognition, ToM, and social skills) through repeated practice and skills-based training. While preliminary evidence supports the effectiveness of targeted non-VR-based social cognitive interventions for autistic adults [22], the existing studies are limited in number and are subject to significant methodological limitations, such as not being randomized clinical trials or having small sample sizes (n≤17) categorizing them as pilot studies. Previous studies of social cognition training for autistic adults typically target multiple domains, such as emotion processing and recognition, ToM, and social perception and attribution. Hypothesized mechanisms of change include repeated practice, gradual and errorless learning, compensatory strategy training, and supportive contexts that promote generalization [22-24]. Specifically for VR-based interventions, additional mechanisms of change include immersion in ecologically valid scenarios, graded exposure, individually tailored training, real-time feedback, control of sensory load, and structured transfer to daily life [25,26].

### VR in Mental Health

The use of VR holds great potential for mental health interventions by enabling researchers and clinicians to evoke psychological reactions to lifelike scenarios in real-time within a controlled laboratory environment. VR serves as a powerful tool for delivering exposure therapy, as it creates a safe, flexible, and controlled space for immersive exposure to challenging situations. This approach allows individuals to practice confronting real-world challenges without facing the consequences of real-life situations, while also reducing the social pressure often associated with face-to-face interactions [27]. As a result, VR exposure is considered an effective tool for treating various psychological challenges, with minimal risks of adverse side effects [28]. Additionally, creating in vivo exposure to challenging social situations in daily life is often difficult to arrange and nearly impossible to control and can be overly stressful for participants, making engagement in such settings challenging. Interventions using VR exposure can overcome these obstacles by offering complete control over various scenarios and their elements, leading to greater user acceptance. As a result, participants may be more inclined to engage in exposure therapy when it is provided in a VR setting [29]. Additionally, VR enables the creation of highly personalized interventions by allowing control over the level of exposure, tailoring scenarios to individual needs, and making real-time adjustments.

### VR-based Interventions for Autistic Adults

By using immersive VR exposure in interventions targeting social cognitive challenges in autistic adults, it becomes possible to assess real reactions and responses to life-like challenging social situations, allowing access to the real-time cognition of the participant in such situations. This approach might be of particular importance for interventions aimed at individuals on the autism spectrum, since self-awareness and self-insight are often compromised by alexithymia (difficulties in identifying and describing one’s own emotional state) [30]. Alexithymia can hinder the accurate assessment of psychosocial difficulties in interventions, as more traditional approaches often require individuals to describe their own emotions retrospectively. Therefore, using VR exposure might enhance assessment accuracy and, in turn, enable more personalized and effective interventions. Furthermore, individuals on the autism spectrum may prefer technology-based interventions [23], and preliminary evidence indicates that VR-based interventions lead to faster symptom reduction than traditional psychological interventions [31,32].

Given that social cognitive differences in autistic adults often involve emotion processing and recognition, ToM, social understanding, and motivation [14,15], VR-based interventions are theoretically well-suited to target these domains. The ability to simulate complex, dynamic social interactions in a controlled, repeatable, and safe environment enables targeted practice of interpreting and responding to social cues, adjusting difficulty as skills improve [24,33]. By providing immediate feedback, opportunities for graded exposure, and the capacity to individualize scenarios, VR can support the gradual enhancement of underlying neuropsychological processes essential for real-world social functioning.

Research on VR-based interventions for individuals on the autism spectrum has gained significant momentum in recent years. Most previous studies have investigated interventions targeting specific functional domains, such as job interviewing skills, driving skills, and adaptive social skills [26,34]. While some studies have examined the impact of VR-based interventions on cognition in individuals on the autism spectrum, these studies have primarily addressed specific neurocognitive domains and daily life skills [35], with comparatively fewer studies investigating social cognition [24,25]. The results of these studies have provided preliminary evidence supporting the feasibility of VR-based interventions for individuals on the autism spectrum. These studies have yielded promising results, indicating positive effects on specific functional and social skills, as well as neurocognitive domains like attention, memory, and executive functioning and social cognitive domains including emotion recognition and ToM. However, the findings are limited by small sample sizes, lack of randomized, clinical trials, and insufficient long-term follow-up. Additionally, most research has focused on children and adolescents. Therefore, large-scale, methodologically rigorous trials are urgently needed to investigate the effectiveness of VR-based interventions for autistic adults to draw more firm conclusions.

### Objectives

The aim is to conduct the hitherto largest clinical randomized trial globally, comparing VR-based social cognitive training (VRSCT) + treatment as usual (TAU) to TAU for autistic adults. The objective is to examine whether VRSCT is effective in improving psychosocial functioning, quality of life, and co-occurring clinical symptoms and to reduce social cognitive deficits in autistic adults. Additionally, we will examine whether the VRSCT is cost-effective.

We hypothesize the following:

1. VRSCT+TAU will be superior to TAU in improving psychosocial functioning, quality of life, co-occurring clinical symptoms, and reducing social cognitive deficits in autistic adults.
2. VRSCT will be cost-effective in improving daily life functioning and reducing social cognitive deficits in autistic adults.

### Trial Design

STEPS (Social Cognitive Training Enhancing Pro-Functional Skills) is a randomized, assessor-blinded, parallel-group superiority clinical trial. A total of 140 participants will be allocated to receive either VRSCT+TAU or TAU alone. Assessments will be carried out at baseline, at cessation of intervention (3 months post baseline), and at 6 months post baseline. A flow diagram of the trial is presented in the Results section. A stratified block randomization with a concealed randomization sequence will be conducted with a 1:1 allocation. Independent assessors, blinded to the treatment, will evaluate outcomes. The analysis of outcomes will be carried out according to the intention-to-treat principle [36].

## Methods

### Study Setting

The trial will enroll participants from public and private outpatient facilities in Denmark that specialize in the clinical assessment and diagnosis of autism in adulthood. The clinical staff at these sites, including nurses, psychologists, doctors, and social workers, will approach relevant participants and refer them to the project group. Additionally, relevant participants from community-based social support services in the Capital Region of Denmark will be referred to the trial by the multidisciplinary staff at these locations. Participants will also be recruited via TrialTree, an online recruitment platform that specializes in connecting interested participants with targeted and relevant clinical research projects. Furthermore, self-referrals will be accepted, provided that the eligibility criteria for participation are met. Assessment, randomization, experimental intervention, and analysis of outcomes will be carried out by the VIRTU Research Group located at Mental Health Center Copenhagen, 2900 Hellerup, Denmark.

### Eligibility Criteria

Participants must provide written, informed consent prior to the initiation of any assessment or intervention in the STEPS trial. Inclusion and exclusion criteria are listed in Textbox 1.

Textbox 1. **Inclusion and exclusion criteria of the STEPS (Social Cognitive Training Enhancing Pro-Functional Skills) trial.**

**Inclusion criteria**

- Age ≥18 years
- Diagnosis of autism (*ICD-10* [*International Statistical Classification of Diseases and Related Health Problems, Tenth Revision*]: F84.0, F84.1, F84.5, and F84.8)
- T-score of ≥60 on the Social Responsiveness Scale Total Score, Second Edition, Adult Form, Self-report

**Exclusion criteria**

- A diagnosis of organic brain disease (*ICD-10*: F00-F09)
- Intellectual disability (IQ <70)
- Inadequate command of spoken Danish or English for engaging in the assessments and intervention
- Co-occurring attention-deficit/hyperactivity disorder with medically untreated symptoms
- Immediate risk of homicide or suicide

The study is conducted in Denmark, where the *ICD-10* (*International Statistical Classification of Diseases and Related Health Problems, Tenth Revision* [37], remains the current standard for diagnostic classification in clinical practice. As no autism diagnostic assessment will be conducted as part of the trial, participants are required to provide documentation confirming a clinical diagnosis of autism based on *ICD-10* criteria (*ICD-10* codes, namely F84.0, F84.1, F84.5, and F84.8). Diagnostic assessments based on autism-specific instruments, for example, Adult Asperger Assessment [38], Autism Diagnostic Observation Schedule, Second Edition (ADOS-2 [39]), and Autism Diagnostic Interview - Revised (ADI-R [40]), preferably supplemented by comprehensive developmental history and information from multiple informants (eg, self-report and informant-report) and from different modalities (eg, observation, interviews, and questionnaires), as recommended for improving autism diagnostic procedure, especially relevant for girls and women [41], are accepted. In cases where a diagnosis was confirmed without using formal assessment tools, documentation will be accepted only if the evaluation was conducted by an experienced clinician and is adequately documented.

To ensure participants experience significant deficiencies in social interactions, the Social Responsiveness Scale, Second Edition, Adult Form, Self-report (SRS-2A) will be used to assess the level of social impairments. A sex-normed T-score of ≥60 on the SRS-2A total score was chosen as the cutoff since a T-score of 60 is the lower threshold for mild deficiencies in social behaviors that are clinically significant and may lead to mild or moderate difficulties in daily social interactions [42]. Estimated full-scale IQ will be estimated using the 2 subtests, Vocabulary and Matrix Reasoning, from the Danish version of the Wechsler Adult Intelligence Scale, Fourth Edition (WAIS-IV) [43], which are known to correlate strongly with full-scale IQ [44]. Participants with co-occurring attention-deficit/hyperactivity disorder (ADHD) must demonstrate a preliminary satisfactory treatment response to ADHD medication at the time of enrollment. This determination will be based on mutual agreement between the participant and the clinician responsible for their ADHD treatment, confirming that targeted ADHD symptoms are well managed. Additionally, no changes in the type or dosage of ADHD medication are permitted within the 4 weeks preceding enrollment in the trial. The type and dosage of ADHD medicine will be thoroughly mapped and documented at enrollment, and at follow-up assessments. Further details of this criterion are displayed in the “Relevant Concomitant Care and Interventions Permitted or Prohibited During the Trial” section. Additionally, general psychopathology will be assessed with the Danish version of the Mini-International Neuropsychiatric Interview (MINI) [45].

### Interventions

#### Explanation for the Choice of Comparator

This trial compares the effects of VRSCT+TAU to TAU alone offered to autistic adults in Denmark. TAU usually includes short-term psychoeducation provided by specialized facilities for autistic adults, following psychiatric assessment and diagnosis. Additionally, TAU often involves supportive counselling or practical assistance in daily life situations, delivered in community settings. The exact content and duration of TAU received by each participant will be mapped and documented upon trial completion, enabling examination of potential between-group differences regarding the content and duration of TAU. This definition of TAU as a comparator was chosen, although its specific components are not strictly defined, because it represents the most common intervention currently offered to autistic adults in Denmark. To accurately assess the efficacy of VRSCT, it is essential to compare it against a standard treatment that represents routine, real-world care provided to this population.

#### Intervention Description

**Figure 1.**
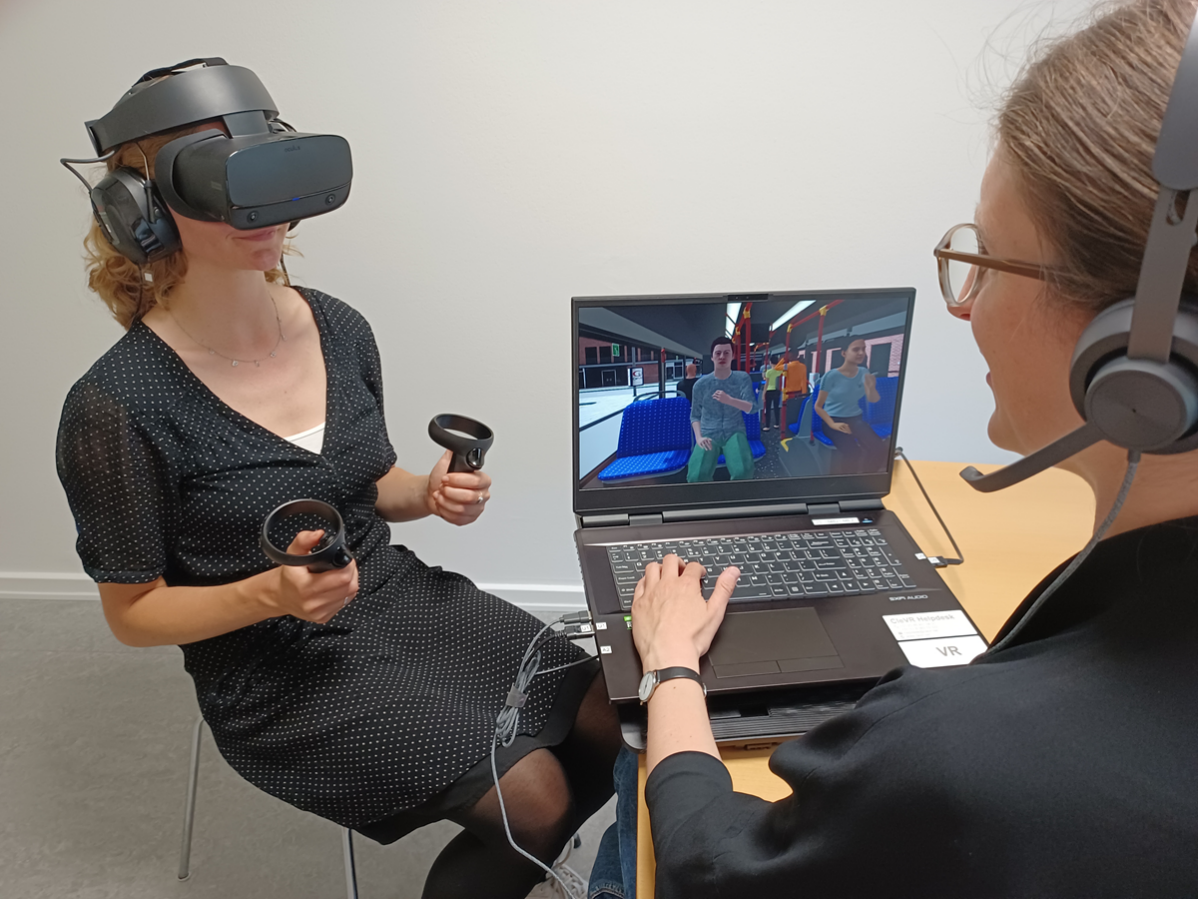
Virtual reality–based social cognitive training in STEPS (Social Cognitive Training Enhancing Pro-Functional Skills); a participant (portrayed by a colleague from the VIRTU Research Group) practices conversing with a stranger during a virtual bus ride.

Participants in the experimental group will receive 12 weekly, 1-hour sessions of VRSCT, with the primary aim of enhancing psychosocial functioning and social cognition through exposure to social environments in VR. Figure 1 displays an example of the VRSCT in STEPS.

Relatives are invited to participate in the intervention. Participation of relatives is dependent on the participant’s consent. Relatives will participate in 1 session at the beginning of the intervention to add information to the assessment of the participants’ challenges. Relatives will also participate in 1 session at the end of the intervention to support transfer to everyday life. If participants decline involvement of relatives, this will be registered, and the intervention will consist of 10 individual sessions. This 12-session format is in line with previous positive findings of other short-term interventions (10‐14 sessions) targeting social cognition in autistic adults [46,47].

The VRSCT in the trial is a manualized cognitive behavioral therapy–based intervention targeting social cognition. The intervention consists of 3 modules, namely emotions, social understanding (including ToM), and complex social interactions. Within these modules, the intervention is highly personalized to meet the specific needs of each participant. The intervention uses the Social Worlds VR software developed by CleVR. Originally developed for psychotic disorders, the Social Worlds software has been adapted to meet the needs of other clinical populations, including personality disorders and autism spectrum conditions [48]. The software enables exposure to different social environments, such as a bus, café, street, supermarket, park, classroom, waiting room, home environment, and an office setting. In these environments, social interactions and role-plays, facilitated by the therapist, can take place. Additionally, the software allows for perspective switching, enabling the therapist to record and replay role-plays the participant engages in. These interactions can be replayed from multiple perspectives (eg, from another person involved or an observer), allowing the participant to experience the situation from someone else’s point of view and thereby stimulate ToM development. By addressing the participant’s individual social interaction challenges through exposure to VR-based social situations and role-plays, key social cognitive functions can be targeted. These include the speed and accuracy of emotion identification in complex, life-like situations, distinguishing relevant from irrelevant social information, and ToM. Additionally, daily social communication and functioning are trained by engaging participants in virtual encounters with avatars (eg, initiating conversations, engaging in small talk, and navigating misunderstandings and setting boundaries). The *S*ocial Worlds software allows for a highly individualized and tailored approach by having various daily life scenarios and 100 different avatars. The highly modifiable social interactions in VR allow for addressing social cognitive impairments across a broad range of severities. During virtual engagements, the therapist facilitates cognitive behavioral therapy–based dialogues to enhance psychosocial functioning, develop new coping strategies, and reduce avoidant behavior, including social isolation. In cocreation with the participant, the therapist facilitates individually tailored dialogues and role-plays, based on the participant’s goals and challenges, and selects the most appropriate avatars in terms of appearance and voice, rather than relying on preprogrammed scenarios or fixed tasks. The VR intervention was co-developed with autistic adults and is continuously tailored to individual goals, sensory needs, and communication styles, supporting skills and confidence in situations that participants themselves find meaningful. In this way, the intervention focuses on the goals of participants rather than enforcing conformity to neurotypical standards.

#### Fidelity

All sessions of VRSCT will be audiotaped, and a selected number of sessions will be rated by an independent rater to ensure fidelity to the treatment protocol. Fidelity will be assessed by an independent rater with clinical expertise in autism, using a manual developed specifically for the STEPS intervention and based on key elements from the Cognitive Therapy Scale - Revised (CTS-R [49]). A total of 10 participant courses will be randomly selected across the recruitment period (3 from the early phase, 4 from the middle phase, and 3 from the late phase) to ensure representation across all trial phases and across therapists delivering the intervention. From each selected course, 3 sessions (one from the early, one from the middle, and one from the late phase of the course) will be randomly chosen for fidelity rating, resulting in 30 rated sessions in total. Time spent in VR will gradually increase during the first sessions and continue to build up across the intervention. The exact time spent in VR will be documented for each session.

#### Comparison Group

Participants in the comparison group will receive TAU alone, which typically comprises short-term psychoeducation provided by the clinical staff at the psychiatric facilities for autistic adults, following psychiatric assessment and diagnosis. Additionally, supportive counselling or practical assistance in daily life situations can be provided in community settings by social workers.

#### Criteria for Discontinuing or Modifying Allocated Interventions

Participants can choose to discontinue the interventions at any given time without any impact on their future psychiatric care. If participants experience a deterioration in their mental health condition during the intervention, this will be considered, and the intervention will be adjusted accordingly. Likewise, follow-up assessments and individual sessions in the intervention may be postponed if participants are unable to attend due to hospitalization. Any psychiatric hospitalization and its extent will be mapped upon trial completion.

#### Strategies to Improve Adherence to Intervention Protocols

Exposure to challenging social environments is a key component of the experimental intervention. Participants may encounter social situations that can provoke discomfort and anxiety. To prevent overwhelming the participant and reduce the risk of dropout, it is essential that the intervention be tailored to each participant’s optimal level of challenge. This ensures that difficult situations are addressed without exceeding their coping abilities. The therapists will maintain this balance through continuous dialogue with participants about their experiences and by promoting adherence to the intervention.

#### Relevant Concomitant Care and Interventions Permitted or Prohibited During the Trial

The prevalence of co-occurring psychiatric diagnoses is considered high among autistic adults, with ADHD (25.7%) being the most prevalent comorbid condition, followed by mood disorders (18.8%) and anxiety disorders (17.8%) [50]. The estimated prevalence of ADHD in the total autistic population is varying widely in the literature. Recent meta-analyses estimate the pooled point prevalence rate of ADHD in the autistic population to range from 28% to approximately 38% [51,52]. To enhance the ecological validity of the trial, co-occurring psychiatric conditions and associated medical treatments will not constitute exclusion criteria apart from medically untreated ADHD. As previously mentioned, participants with co-occurring ADHD must demonstrate a preliminary satisfactory treatment response to ADHD medication at the time of enrollment. This determination will be based on mutual agreement between the participant and the clinician responsible for their ADHD treatment, confirming that targeted ADHD symptoms are well managed. Additionally, no changes in the type or dosage of ADHD medication are permitted within the 4 weeks preceding enrollment in the trial. This criterion is essential due to the relatively high prevalence of co-occurring ADHD in individuals on the autism spectrum. Further, many adults are often being assessed for (and diagnosed with) ADHD while undergoing evaluation for autism. As a result, many potential participants may be titrating ADHD medication while being referred to the trial. To be able to effectively distinguish the effects of the trial intervention from those of ADHD medication, this criterion is essential. Furthermore, all psychopharmacological treatments will be thoroughly mapped and documented upon trial completion.

#### Provisions for Ancillary and Posttrial Care

All participants have access to the Danish public health care system before, during, and after participation in the trial. The experimental intervention is considered an add-on treatment to the standard treatment. If posttrial care is needed, it will be provided by the health care system and will follow national standards. If participants experience a significant deterioration in their mental health during the trial, the project may initiate a referral for outpatient psychiatric follow-up and treatment. Additionally, participants in the trial are covered by the Danish Patient Compensation Association, which evaluates and covers injuries resulting from health care treatment.

### Outcomes

Table 1 provides an overview of the outcome measures and their assessment time points.

**Table 1.** Schedule of enrollment, intervention, and assessments

Content	Enrollment	Baseline assessment	Allocation	Intervention	Follow-up assessment 3 months post baseline	Follow-up assessment 6 months post baseline
Time points		t_0_			t_1_	t_2_
Information about the trial	✓					
Informed consent	✓					
Eligibility evaluation	✓					
SRS-2A^a^	✓					
Vocabulary and Matrix Reasoning (WAIS-IV^b^)	✓					
MINI^c^	✓					
Randomization			✓			
Interventions						
Comparison group (TAU^d^)				✓		
Experimental group (VRSCT^e^+TAU^d^)				✓		
Assessments						
Primary outcome						
SRS-2A^a^		✓ (administered at enrollment)			✓	✓
Secondary outcomes						
HiSoC^f^		✓			✓	✓
SRS-2AI^g^		✓			✓	✓
SIAS^h^		✓			✓	✓
ERT^i^ from CANTAB^j^		✓			✓	✓
EQ^k^		✓			✓	✓
Exploratory outcomes						
PSP^l^		✓			✓	✓
BRIEF-A^m^		✓			✓	✓
GSE^n^		✓			✓	✓
WHO-5^o^		✓			✓	✓
EQ-5D^p^		✓			✓	✓
SR-AS^q^		✓				
RBQ-3^r^		✓				
GAFS-8^s^		✓			✓	✓
CAT-Q^t^		✓			✓	✓
ASRS^u^		✓			✓	✓
BDI-II^v^		✓			✓	✓
TASIT-S^w^		✓			✓	✓
DACOBS^x^		✓			✓	✓
SSPA^y^		✓			✓	✓
Other measures						
CSQ^z^					✓	✓
NEQ^aa^					✓	✓
MPS-MSDV^ab^ (Only experimental group, 3 and 10 weeks post baseline)				✓		
SSQ^ac^ (Only experimental group, 3 and 10 weeks post baseline)				✓		
RTQ^ad^ (Only experimental group, 1 week post baseline)				✓		
I-VAS^ae^ (Only experimental group, 12 weeks post baseline)				✓		

a SRS-2A: Social Responsiveness Scale, Second Edition, Adult Form, Self-report.

b WAIS-IV: Wechsler Adult Intelligence Scale, Fourth Edition.

c MINI: Mini-International Neuropsychiatric Interview.

d TAU: treatment as usual.

e VRSCT: virtual reality-based social cognitive training.

f HiSoC: High-Risk Social Challenge task.

g SRS-2AI: Social Responsiveness Scale, Adult Form, Informant report.

h SIAS: Social Interaction Anxiety Scale.

i ERT: Emotion Recognition Task.

j CANTAB: Cambridge Neuropsychological Test Automated Battery.

k EQ: Empathy Quotient.

l PSP: Personal and Social Performance Scale.

m BRIEF-A: Behavior Rating Inventory of Executive Function - Adult Version, Self-Report.

n GSE: General Self-Efficacy Scale.

o WHO-5: WHO-5 Well-Being Index.

p EQ-5D: EuroQOL Five Dimensions Questionnaire.

q SR-AS: Sensory Reactivity in Autism Spectrum questionnaire .

r RBQ-3: Repetitive Behavior Questionnaire-3.

s GAFS-8: General Alexithymia Factor Score.

t CAT-Q: Camouflaging Autistic Traits Questionnaire.

u ASRS: Adult ADHD Self-Rating Scale.

v BDI-II: Beck Depression Inventory-II.

w TASIT-S: The Awareness of Social Inference Test short form.

x DACOBS: Davos Assessment of the Cognitive Biases Scale.

y SSPA: Social Skills Performance Assessment.

z CSQ: Client Satisfaction Questionnaire.

aa NEQ: Negative Effects Questionnaire.

ab MPS-MSDV: Multimodal Presence Scale, Modified Shortened Danish Version.

ac SSQ: Simulator Sickness Questionnaire.

ad RTQ: Readiness for Therapy Questionnaire.

ae I-VAS: Insight - Visual Analog Scale

#### Primary Outcome

The primary outcome is the change of social impairment from baseline to intervention cessation, measured at 3-month follow-up. At follow-up assessments, the observation period will comprise the last 2 weeks, instead of the previous 6 months, as at baseline assessment. The SRS-2A will be used to measure the level of social impairment [42]. The SRS-2A is a 65-item self-report rating scale designed to assess social impairments related to autism spectrum conditions [53]. SRS-2A consists of 65 items, scored on a 4-point Likert Scale (0‐3). Each item is scaled from 0 (never true) to 3 (almost always true), generating a total score ranging from 0 to 195. The SRS-2A provides a continuous measure of social impairment, where a higher score reflects a greater degree of difficulties with social interactions in everyday life. The SRS-2 has been extensively used in autism research across different age groups and has demonstrated strong validity and reliability in both children [54,55] and adults [56-58].

#### Secondary Outcomes

Secondary outcomes will assess changes in performance-based social skills, informant-reported social functioning, social anxiety, emotion recognition, and empathy:

1. Social skills performance will be measured using the High-Risk Social Challenge (HiSoC) task, a standardized performance-based task assessing social interpersonal skills [59]. HiSoC scoring comprises 3 primary factors, namely affect, odd behavior and language, and social interpersonal skills. Additionally, an overall impression of the performance is rated. These 3 factors and the general impression are added to calculate the HiSoC total score.
2. Social impairment will be measured using the Social Responsiveness Scale, Adult Form, Informant report (SRS-2AI) [42]. The SRS-2AI, a 65-item rating scale, assesses the level of social impairment based on informant observation. Items are rated on a 4-point Likert Scale, from 0 (never true) to 3 (almost always true), generating a total score range of 0-195. The items, while similar in content to the self-report version, are adapted for the informant’s perspective. The SRS-2AI provides a continuous measure of social impairment, with higher scores indicating more severe difficulties with social functioning in everyday life.
3. Social anxiety levels will be measured by the Social Interaction Anxiety Scale (SIAS) [60]. The SIAS consists of 20 items rated on a 5-point Likert scale, from 0 (not at all characteristic of me) to 4 (extremely characteristic of me). Items 5, 9, and 11 are positively worded items and require reverse scoring. The total SIAS, ranging from 0 to 80*,* is calculated by summing all item ratings, with higher scores indicating greater social anxiety experienced in social interaction*.*
4. Emotion recognition ability will be assessed using the Emotion Recognition Task (ERT) from the Cambridge Neuropsychological Test Automated Battery (CANTAB) [61]. The ERT outcome measures include the percentage and count of correctly and incorrectly identified emotions displayed in images of facial expressions, as well as overall response latencies. These measures can be assessed across individual emotions or as an aggregate across all emotions.
5. The level of empathy will be assessed using the Empathy Quotient (EQ), a 60-item questionnaire designed to measure empathy in adults with normal intelligence [62]. Each item is scored on a 4-point scale (strongly agree, slightly agree, slightly disagree, and strongly disagree), yielding a possible score from 0 to 80, with higher scores reflecting higher levels of empathy.

#### Exploratory Outcomes

##### Overview

Below, exploratory outcomes are grouped into the following categories: psychosocial functioning and quality of life, clinical symptoms, social cognition and performance, and other measures.

##### Psychosocial Functioning and Quality of Life

This will be measured using the following outcomes: (1) the Personal and Social Performance scale (PSP) [63] to assess daily life social functioning; (2) the Behavior Rating Inventory of Executive Function - Adult Version, Self-Report (BRIEF-A) [64,65] to assess everyday executive function; (3) the General Self-Efficacy Scale (GSE) [66] to assess perceived self-efficacy; (4) WHO-5 Well-Being Index (WHO-5) [67] to assess subjective, psychological well-being; and (5) the EuroQOL 5D questionnaire (EQ-5D) [68,69] to assess health-related quality of life and to conduct cost-effectiveness analysis.

##### Clinical Symptoms

This will be measured using the following outcomes: (1) Sensory Reactivity in Autism Spectrum (SR-AS) questionnaire [64] to assess sensory reactivity; (2) the Repetitive Behavior Questionnaire-3 (RBQ-3) [70] to assess restricted and repetitive behaviors and interests; (3) the General Alexithymia Factor Score (GAFS-8) [71] to assess alexithymia; (4) the Camouflaging Autistic Traits Questionnaire (CAT-Q) [72] to assess social camouflaging behaviors; (5) the Adult ADHD Self-Report Scale (ASRS) [73] to assess ADHD symptoms; and (6) the Beck Depression Inventory-II (BDI-II) [74] to assess depressive symptoms.

##### Social Cognition and Performance

This will be measured by the following outcomes: (1) the Awareness of Social Inference Test short form (TASIT-S), part 2 Social Inference, Minimal) [75,76] to assess ToM and complex emotion comprehension; (2) the Davos Assessment of the Cognitive Biases Scale (DACOBS) [77] to assess cognitive biases; and (3) the Social Skills Performance Assessment (SSPA) [78] to assess social communication skills (competence and appropriateness of conversational skills).

##### Other Measures

These measures related to participants’ experiences of the intervention will be assessed using the following measures: (1) the Client Satisfaction Questionnaire (CSQ) [79] to assess satisfaction with the assigned intervention; the Negative Effects Questionnaire (NEQ) [80] to assess negative events and outcomes; (2) the Multimodal Presence Scale, Modified Shortened Danish Version (MPS-MSDV) [81] to assess involvement and immersion into the VR environments (only administered to participants in the experimental group); (3) the Simulator Sickness Questionnaire (SSQ) [82] to assess simulator sickness related to being in VR (only administered to participants in the experimental group); (4) the Readiness for Therapy Questionnaire (RTQ) [83] to assess attitudes and readiness for therapy aimed at resolving problems; and (5) an Insight-Visual Analog Scale (I-VAS) to assess level of insight into challenges related to social interaction. Participants and therapists will indicate, on a scale from 1 to 10, the extent to which the intervention has increased the participant’s awareness of their challenges with social interaction, with 1 representing no increased awareness and 10 indicating the highest possible increase in awareness. I-VAS will be administered by the therapists in the intervention and only to participants in the experimental group upon completion of the intervention.

### Sample Size and Power Calculation

The sample size calculation is based on detecting a between-group difference in the primary outcome, the SRS-2A, at cessation of the intervention (3 months post baseline). Previous studies have reported considerable variation in the SD of the social responsiveness scale total score when assessing social impairment in autistic adults [25,56,58]. As a result, we have not been able to determine the minimal clinically important difference. Therefore, we have based our sample size calculation on a medium effect size (Cohen *d*=0.5), which corresponds to a difference of 0.5 SD between the experimental group (VRSCT+TAU) and the comparison group (TAU). With 80% power and a 2-sided significance level of α=.05, 63 participants are required in each group to detect this difference. To account for an anticipated 10% dropout rate, we plan to recruit a total of 140 participants. Sample size calculation is based on a medium effect size (Cohen *d*=0.5) in accordance with established practice in clinical trial design. However, it may not reflect the smallest change considered clinically meaningful. Clinical significance will therefore be interpreted alongside observed effect sizes and CIs in subsequent reporting to ensure meaningful interpretation of trial outcomes.

### Recruitment

The project group has established strong collaborations with several public and private outpatient facilities in Denmark, which are specializing in the clinical assessment and diagnosis of autism in adulthood. Based on the collaboration agreement, the multidisciplinary staff at these sites will routinely approach relevant participants and refer them to the VIRTU Research Group. To foster collaboration and ensure ongoing referrals to the trial, staff from the VIRTU Research Group will regularly attend conferences and meetings at the various recruitment sites and invite clinicians to discuss potential participants.

### Allocation

#### Sequence Generation

Participants who provide signed informed consent and meet the eligibility criteria will be randomly allocated to receive either TAU or VRSCT+TAU upon completion of the baseline assessment. Randomization will be performed using a centralized, automated, computer-generated randomization module in the online REDCAP (Research Electronic Data Capture; Vanderbilt University) system (refer to “Data Management” section). A stratified block randomization with a concealed randomization sequence will be used, with block sizes that are variable and unknown to the investigators. Randomization will be stratified by site and sex. The allocation of the randomized intervention will remain concealed to investigators and assessors until the statistical analyses of outcome data have been completed.

#### Concealment Mechanism

When a participant has completed the baseline assessment, the trial assessor, who is responsible for conducting the assessment, will email one of the trial therapists responsible for generating the allocation sequence. The assessor will then receive a confirmation email acknowledging receipt only. The trial therapist has exclusive access to the randomization module in REDCAP (refer to “Data Management” section), ensuring that assessors are blinded to intervention allocation.

#### Implementation

Allocation sequences are generated by one of the trial therapists. Enrollment and assignment to the interventions are carried out by the therapists who are conducting the interventions in the experimental group.

#### Blinding

Following the allocation to one of the interventions, the outcome assessors will be blinded to the participants’ group assignment. Due to the nature of the intervention in the experimental group (VRSCT), participants, therapists conducting the intervention, and the clinicians managing TAU will not be blinded to the received interventions. Participants will be instructed prior to follow-up assessments not to disclose their allocation to the outcome assessors. To maintain blinding, assessments and interventions will be conducted at separate locations, ensuring that the assessors remain unaware of group assignment. Assessors will be instructed to notify their supervisors immediately if they believe blinding has been broken. If unblinding of the outcome assessor occurs, another outcome assessor blind to the allocation of intervention will conduct the subsequent assessments.

### Data Collection

#### Assessment and Collection of Outcomes

Assessments will be conducted at baseline (t_0_), 3 months post baseline (t_1_), and 6 months post baseline (t_2_). An overview of assessments and outcome measures is provided in Table 1. Outcome assessors will be psychologists who will receive comprehensive training before conducting assessments. To ensure consistency and maintain data quality, assessors will participate in monthly supervision sessions focused on the administration and scoring of assessments. The following rater-based assessments, HiSoC, PSP, and SSPA, will be video- and audio-recorded to enable subsequent scoring. To ensure high interrater reliability, a structured calibration procedure has been implemented. First, the initial 20 consecutive assessments of each rater-based measure will be scored through consensus by the 2 outcome assessors together with a senior researcher and clinical expert in autism. This process is used to establish a shared understanding of scoring criteria and interpretation. Subsequently, the next 10 consecutive assessments will be independently rated by both outcome assessors, and intraclass correlation coefficients will be calculated to evaluate interrater agreement. Provided that acceptable reliability is achieved (intraclass correlation coefficient ≥0.75) [84], the assessors will continue to rate independently. To prevent rater drift and ensure continued data quality throughout the trial, monthly consensus rating sessions will be conducted involving both assessors and the senior researcher. Further, the assessment of co-occurring psychopathology using the MINI will be scored through consensus. Diagnoses based on the MINI will be diagnosed according to both *ICD-10* and *ICD-11* (*International Classification of Diseases, Eleventh Revision*) criteria. All training, supervision, and reliability procedures will be overseen by a senior researcher and clinical expert in autism.

#### Plans to Promote Participant Retention and Complete Follow-Up

To enhance participant retention and ensure follow-up assessments, several initiatives will be taken. Participants will be contacted by phone approximately 1 week before the planned follow-up assessments, and travel expenses for public transportation will be compensated to reduce the financial burden associated with participation in the trial. Additionally, assessors can rearrange scheduled assessment times to accommodate participants’ needs. In cases where participants leave the trial prematurely, efforts will be made to complete follow-up assessments. If participants are unable to complete the full assessment battery, priority will be given to obtaining the primary and secondary outcomes.

### Data Management

Data from evaluation of eligibility and assessments during the trial will be entered directly into the electronic case report form using the data entry system REDCAP. REDCAP is an electronic data capture tool hosted by the Center for IT, Medical Technology and Telephony (CIMT) in the Capital Region of Denmark. When electronic entry is not possible, data will be collected on paper and later transferred into REDCAP. Data on paper will be stored locally and secured. Using REDCAP, surveys can securely be distributed and returned via E-boks, a public Danish digital mailing system that all citizens possess, reducing the risk of data loss and unauthorized access. Data for each participant will be linked to a unique serial number, with only assigned researchers having access to REDCAP. REDCAP includes a complete audit trail of data transactions, detailed user rights, and access control management, ensuring compliance with Danish legislation (the Act on Processing Personal Data). Research data will be exported from REDCap without personal identifiers. Data will be exported to well-known software packages (eg, SPSS [IBM Corp], SAS [SAS Institute], Stata [StataCorp LLC], and R [R Foundation for Statistical Computing]) and placed in logged folders on a network drive under the control of the CIMT. A data manager will ensure that all variables are properly defined with variable and value labels. All derived variables will be properly defined, and algorithms will be kept in special files. All data will be examined carefully to identify errors in data entry.

### Statistical Analysis of Primary and Secondary Outcomes

Analyses will be performed on the intention-to-treat population for the primary and secondary end points, that is, analyzing all randomized participants according to the arm they were randomized to. The analysis method will be ANCOVA (analysis of covariance) of change from baseline with treatment as a factor and baseline value as a covariate at the corresponding postbaseline visit, that is, third month. Additionally, stratification variables and baseline values of social anxiety, alexithymia, and social function will be included as covariates. Treatment difference point estimates will be reported together with 95% CIs and *P* value. Missing data will be handled using multiple imputation. For the exploratory end points, a modified intention-to-treat set will be used, requiring at least 1 postbaseline measurement for the inclusion in the analysis for each end point. The analysis method will be the linear mixed effects model. Results will be reported for both postbaseline visits. Details of model specifications, imputation methods and assumptions, sensitivity analyses, and subgroup analyses will be provided in the forthcoming statistical analysis plan, if applicable. Results will be reported with a 95% CI and *P* value, using a 5% significance level. Baseline demographic and clinical characteristics will be summarized for each group, using descriptive statistics (eg, means, SDs, medians, and ranges for continuous variables and frequencies and proportions for categorical) to assess comparability between groups at baseline. Efficacy analysis will be conducted by a blinded and independent statistician with no contact with the participants in the trial. Reporting will follow CONSORT (Consolidated Standards of Reporting Trials) guidelines [85].

To address potential confounding from changes in psychotropic medication, we will conduct sensitivity analyses to explore whether medication changes influence the primary and secondary outcomes. Medication type and dosage are documented at both follow-up time points (3 and 6 months post baseline). To account for variability in TAU, we document the type, frequency, and duration of TAU received during two predefined periods (1) from enrollment to the 3-month follow-up and (2) from 3- to 6-month follow-up. If systematic between-group differences in TAU exposure emerge, summary TAU variables (eg, number of sessions and service type) will be included as covariates in a sensitivity analysis to adjust for potential confounding. Additionally, exploratory analyses may be conducted to examine whether specific aspects of TAU moderate the intervention effect. In addition, exploratory subgroup analyses will be conducted to examine whether selected baseline characteristics moderate the effect of the intervention on primary and secondary outcomes. Analyses will be based on several prespecified variables, including sex, alexithymia, social anxiety, depression, and ADHD.

### Data Monitoring and Oversight

#### Trial Oversight and Management

The trial will be overseen by a Project Management Group (PMG). The PMG will comprise the principal investigator of the study, who is also the trial manager and daily leader of the trial, the principal therapist of the trial, and the supervisor of the assessors. The PMG will hold monthly meetings throughout the recruitment period. There will be no data monitoring committee. The PMG will oversee trial safety and the status of recruitment and retention.

#### Adverse Events

Although VR interventions are generally well tolerated with minimal to no severe side effects or adverse events [28], including among autistic adults [25], some individuals may still experience cybersickness, which is not considered to be serious. Therefore, we do not expect to encounter any serious side effects or adverse events. Side effects and adverse events will be monitored and recorded throughout the study, as well as any complaints about the intervention. The following are considered as adverse events: (1) hospital admissions, (2) suicide attempts, (3) any violent incident necessitating police involvement (whether survivor or accused), (4) self-harming behavior, and (5) all deaths. Any adverse events will be reported to the Committee on Health Research Ethics of the Capital Region Denmark.

#### Auditing Trial Conduct

The leading members of the PMG will continuously ensure that the trial is conducted according to protocol, that data is collected accurately and reliably, and that the safety and rights of the participants are protected. Any necessary revisions to the processes will be discussed at the monthly PMG meetings.

### Ethical Considerations

#### Research Ethics Approval

The study was approved by the Committee on Health Research Ethics in the Capital Region of Denmark (Project ID H-23055504) and by the Danish Data Protection Agency at the Capital Region of Denmark (project ID p-2023‐14488).

#### Protocol Amendments

Protocol amendments must be approved by the Committee on Health Research Ethics of the Capital Region Denmark. Any modifications to the protocol will be documented in the trial registration record (NCT06438536).

#### Consent

Trained psychologists from the VIRTU Research Group will obtain signed informed consent for participation in the STEPS trial. Referred participants will initially receive information about the trial written in layman’s terms, covering the trial’s background, objectives, design, intervention, randomization process, risks, confidentiality, and legal rights. Next, an appointment will be arranged with a trial researcher to ensure participants understand the information provided and accept random assignment to either VRCST+TAU or to TAU alone. If they decide to proceed, they will sign a consent form confirming they have received adequate written and verbal information about the trial’s purpose, method, benefits, and risks, and agree to participate. Written, informed consent to participate will be obtained from all participants, and participants have the right to withdraw from the study at any time and for any reason. Written information about the trial aimed at the participants and the informed consent form is available from the corresponding author.

#### Privacy and Confidentiality

This protocol does not include any personal data or identifying images of participants, nor will such data be included in any subsequent publications reporting the trial results. All data presented in future papers will be fully anonymized to ensure participant confidentiality.

#### Dissemination

The results of the trial will be shared with the scientific community through presentations at national and international conferences. The findings will also be published in scientific papers submitted to relevant academic journals. Additionally, results will be communicated to trial participants. The implications of the results will be discussed with health care professionals and other stakeholders at clinical conferences. Furthermore, the findings will be presented to the public through meetings with interest groups and to the wider public at local meetings or seminars.

#### Compensation Details

Participants will not receive financial compensation. However, transportation expenses will be reimbursed, and meals will be provided on assessment days to ensure participant comfort and minimize any financial burden associated with participation.

## Results

**Figure 2.**
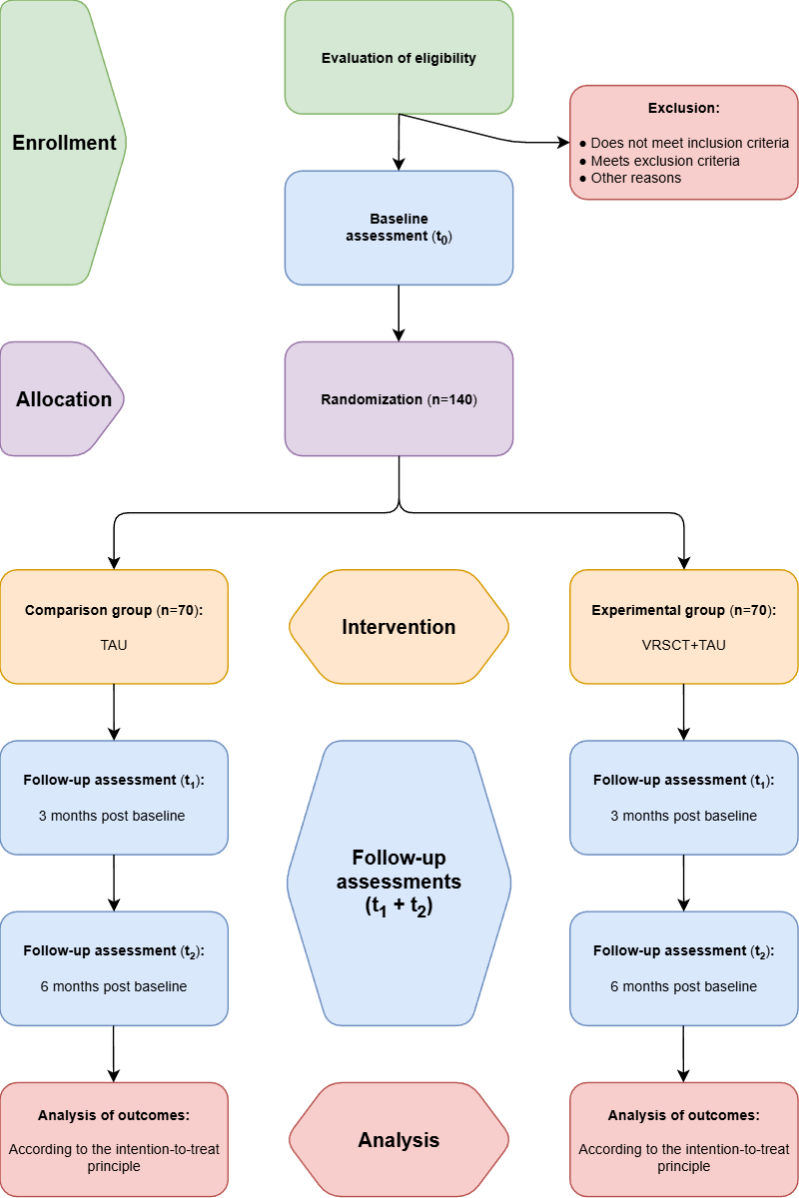
Flow diagram of the STEPS (Social Cognitive Training Enhancing Pro-Functional Skills) trial. TAU: treatment as usual; VRSCT: virtual reality-based social cognitive training.

Funding was secured in October and November 2022. The first participant was enrolled in May 2024. Figure 2 provides a flow diagram of the trial. As of February 2025 (initial manuscript submission), 34 participants had been enrolled, increasing to 97 participants as of December 2025. Completion of enrollment is expected in April 2026. Data analysis is expected to begin in October 2026 following the final 6-month follow-up assessment. Results are anticipated in December 2026 and will be disseminated at international conferences and through peer-reviewed publications by summer 2027.

## Discussion

### Anticipated Findings and Implications

Autistic adults constitute a growing and largely overlooked population, for whom few clinical and research resources have been allocated. Due to the limited evidence on effective interventions for autistic adults, no national clinical guidelines have been developed for psychological interventions aimed at this population in Denmark. Sparse evidence from profunctional studies conducted in England or Sweden is often referenced when attempting to guide psychological treatment approaches for this group [86]. To our knowledge, STEPS is the hitherto largest randomized clinical trial globally investigating the effect of VRSCT for autistic adults. The results of this innovative intervention approach may significantly advance research in the field of autism. If the VRSCT intervention in the STEPS trial proves effective, it has the potential to improve psychosocial functioning and quality of life, co-occurring clinical symptoms, and reduce social cognitive deficits in autistic adults. The intervention was co-developed in collaboration with autistic adults to ensure relevance and alignment with neuro-affirming principles. It is tailored to each participant’s goals, sensory needs, and communication preferences and aims to enhance comfort, confidence, and agency in social situations that participants themselves identify as meaningful. Rather than imposing neurotypical norms of interaction, the approach supports self-defined outcomes and promotes the transfer of skills into everyday life in ways that respect and value neurodivergent ways of relating. Establishing evidence-based interventions is essential to address the debilitating psychosocial challenges encountered by this population, especially considering the absence of established gold-standard interventions. Enhancing profunctional psychosocial skills can significantly impact the lives of these individuals by enabling them to build more meaningful and intimate relationships, better maintain personal and professional relations, and improve educational and occupational functioning. Developing effective interventions for autistic adults can further contribute to filling the critical gap in available support and thereby contribute to alleviating the personal and social consequences and the economic impact associated with autism. If proven effective, the manualized VRSCT intervention from this study could be implemented in mental health services across Denmark and potentially on an international scale, supported by training and ongoing supervision. Additionally, the intervention could be adapted to fit the needs of other clinical populations experiencing social cognitive deficits, such as autistic adolescents, individuals with bipolar disorder, or individuals with psychosis [87]. This could help address the increasing demand for novel, effective interventions for psychiatric conditions globally.

### Limitations

In this trial, eligibility was based on a clinical diagnosis of autism according to *ICD-10* criteria. Although widely used in clinical and research settings, the diagnostic criteria in *ICD-10* and *DSM-5-TR* have been criticized for overemphasizing observable behaviors and reflecting a prototypical male presentation of autism. These limitations can obscure how autism manifests differently across sex and age and may overlook how traits are masked by compensatory strategies, potentially contributing to the underdiagnosis of autism in females and the delayed identification of autism in adults [41,88]. While this may also affect the representativeness of the study sample by leading to fewer females being included, the final sex distribution will help determine the extent to which this concern is reflected in the sample.

The participants in the comparison group will receive TAU, administered across multiple sites. It is expected that there will be variations in the content and duration of TAU among participants. Although the specific details of TAU for each participant will be documented at the end of the trial, this variability remains a limitation. The inconsistency in TAU introduces less control compared to an active control uniformly administered in the same setting as the experimental intervention. However, TAU was chosen as the comparator because it reflects current clinical practice available for autistic adults in Denmark. Additionally, there is no established “gold standard” intervention specifically targeting social cognitive deficits in autistic adults, which otherwise could have been an obvious choice of comparator. Further, given the absence of a “gold standard” comparator and the choice of TAU as the control condition, it is important to note that the immersive and novel nature of the VR intervention may contribute to increased participant expectancy in the intervention group compared with TAU, which does not involve comparable technology. While a sham or low-dose VR control condition was considered to address this technology-expectancy bias, it was not feasible within the scope and resources of this study. To mitigate this risk, we included both a blinded, performance-based outcome measure (HiSoC) and an informant-rated version of the Social Responsiveness Scale (SRS-2AI) as secondary outcomes. This approach allows for triangulation across self-report, informant-report, and blinded assessment. Due to the exclusion criteria regarding autistic adults with medically untreated ADHD and autistic adults with intellectual disability, the results from this trial may not be generalizable to these populations. However, the findings may still provide useful indications of the potential effects for autistic adults with ADHD, and the intervention could eventually be adapted for autistic adults with intellectual disability. Finally, as the present trial does not include follow-up assessment beyond 6 months, the absence of longer-term follow-up (eg, at 12 months) limits conclusions regarding sustained effects. As the effects of VR-based interventions may diminish over time, and follow-up assessments beyond 3 months are often lacking [89], future trials should address this.

### Conclusion

Autistic adults constitute a growing and largely overlooked population with limited evidence-based interventions available. To our knowledge, STEPS is the largest randomized clinical trial globally investigating the effect of VRSCT for autistic adults. If proven effective, VRSCT has the potential to improve psychosocial functioning and quality of life and reduce co-occurring clinical symptoms and social cognitive deficits in autistic adults, thereby potentially contributing to filling the critical gap in available support. The results of the trial should be interpreted in light of key limitations, including heterogeneity in TAU, potential technology-related expectancy effects, and follow-up limited to 6 months.

In light of these considerations, the findings of the trial are expected to advance research in the field of autism and may inform future clinical practice and subsequent trials, including in other clinical populations experiencing social cognitive challenges.

## Supplementary material

10.2196/72854 Checklist 1 SPIRIT checklist.

## Abbreviations

AAA: Adult Asperger Assessment
ADHD: attention-deficit/hyperactivity disorder
ADI-R: Autism Diagnostic Interview - Revised
ADOS-2: Autism Diagnostic Observation Schedule, Second Edition
ANCOVA: analysis of covariance
ASRS: Adult ADHD Self-Report Scale
BDI-II: Beck Depression Inventory-II
BRIEF-A: Behavior Rating Inventory of Executive Function - Adult Version, Self-Report
CANTAB: Cambridge Neuropsychological Test Automated Battery
CAT-Q: Camouflaging Autistic Traits Questionnaire
CIMT: Center for IT, Medical Technology and Telephony
CONSORT: Consolidated Standards of Reporting Trials
CSQ: Client Satisfaction Questionnaire
CTS-R: Cognitive Therapy Scale - Revised
DACOBS: Davos Assessment of Cognitive Biases Scale
*DSM-5-TR*: *Diagnostic and Statistical Manual of Mental Disorders, Fifth Edition, Text Revision*
EQ: Empathy Quotient
ERT: Emotion Recognition Task
GAFS-8: General Alexithymia Factor Score
GSE: General Self-Efficacy Scale
HiSoC: High-Risk Social Challenge task
I-VAS: Insight-Visual Analog Scale
*ICD-10*: *International Statistical Classification of Diseases and Related Health Problems, Tenth Revision*
*ICD-11*: *International Classification of Diseases, Eleventh Revision*
MINI: Mini-International Neuropsychiatric Interview
MPS-MSDV: Multimodal Presence Scale, Modified Shortened Danish Version
NEQ: Negative Effects Questionnaire
PMG: Project Management Group
PSP: Personal and Social Performance Scale
RBQ-3: Repetitive Behavior Questionnaire-3
REDCAP: Research Electronic Data Capture
RTQ: Readiness for Therapy Questionnaire
SIAS: Social Interaction Anxiety Scale
SR-AS: Sensory Reactivity in Autism Spectrum
SRS-2A: Social Responsiveness Scale, Second Edition, Adult Form, Self-report
SRS-2AI: Social Responsiveness Scale, Adult Form, Informant report
SSPA: Social Skills Performance Assessment
SSQ: Simulator Sickness Questionnaire
STEPS: Social Cognitive Training Enhancing Pro-Functional Skills
TASIT-S: The Awareness of Social Inference Test - Short Form
TAU: treatment as usual
ToM: theory of mind
VR: virtual reality
VRSCT: virtual reality-based social cognitive training
WAIS-IV: Wechsler Adult Intelligence Scale, Fourth Edition
WHO-5: WHO-5 Well-Being Index
